# A Modified Protocol with Improved Detection Rate for Mis-Matched Donor HLA from Low Quantities of DNA in Urine Samples from Kidney Graft Recipients

**DOI:** 10.1371/journal.pone.0166427

**Published:** 2016-11-18

**Authors:** Janette Kwok, Leo C. W. Choi, Jenny C. Y. Ho, Gavin S. W. Chan, Maggie M. Y. Mok, Man-Fei Lam, Wai-Leung Chak, Au Cheuk, Ka-Foon Chau, Matthew Tong, Kwok-Wah Chan, Tak-Mao Chan

**Affiliations:** 1 Division of Transplantation and Immunogenetics, Department of Pathology,Queen Mary Hospital, Hong Kong SAR, China; 2 Division of Anatomical Pathology, Department of Pathology, Queen Mary Hospital, Hong Kong SAR, China; 3 Department of Medicine, The University of Hong Kong, Hong Kong SAR, China; 4 Department of Medicine, Queen Elizabeth Hospital, Hong Kong SAR, China; 5 Department of Medicine & Geriatrics, Princess Margaret Hospital, Hong Kong SAR, China; 6 Department of Pathology, The University of Hong Kong, Hong Kong SAR, China; University of Edinburgh MRC Centre for Inflammation Research, UNITED KINGDOM

## Abstract

**Background:**

Urine from kidney transplant recipient has proven to be a viable source for donor DNA. However, an optimized protocol would be required to determine mis-matched donor HLA specificities in view of the scarcity of DNA obtained in some cases.

**Methods:**

In this study, fresh early morning urine specimens were obtained from 155 kidney transplant recipients with known donor HLA phenotype. DNA was extracted and typing of HLA-A, B and DRB1 loci by polymerase chain reaction-specific sequence primers was performed using tailor-made condition according to the concentration of extracted DNA.

**Results:**

HLA typing of DNA extracted from urine revealed both recipient and donor HLA phenotypes, allowing the deduction of the unknown donor HLA and hence the degree of HLA mis-match. By adopting the modified procedures, mis-matched donor HLA phenotypes were successfully deduced in all of 35 tested urine samples at DNA quantities spanning the range of 620–24,000 ng.

**Conclusions:**

This urine-based method offers a promising and reliable non-invasive means for the identification of mis-matched donor HLA antigens in kidney transplant recipients with unknown donor HLA phenotype or otherwise inadequate donor information.

## Introduction

Approximately two-thirds of all kidney transplant recipients in Hong Kong underwent transplantation overseas [1]. The lack of donor information hinders the investigation for donor-specific antibody (DSA) and hampers the diagnosis and management of antibody-mediated rejection. The information is also critical for the future selection of donors in patients who undergo re-transplantation. Donors sharing HLA antigens with previously failed transplants, even in the absence of DSA in the potential recipient, are avoided in some centres [2].

To tackle this problem, we previously reported a novel method to determine mismatched donor HLA using fresh or formalin-fixed paraffin-embedded renal allograft tissue [3,4]. We demonstrated that the allograft tissue expressed both donor and recipient HLA, and mismatched donor antigens could be deduced by subtracting the recipient’s HLA from the allograft HLA. The information facilitates molecular diagnosis and patient management of antibody mediate rejection, and enables appropriate selection of donor kidney for re-transplantation.

Unlike obtaining patient HLA typing from the DNA extracted from peripheral blood samples, donor genetic information could only be obtained either from the kidney allograft or the urine sample. In the present study, the feasibility of the same approach for deduction of mismatched donor HLA from recipients’ urine samples was evaluated. We aimed to circumvent the need of deducing the donor HLA typing by the invasive allograft biopsy procedure and develop a non-invasive and feasible protocol with superior practicality and sensitivity. We launched a territory-wide study to investigate whether donor and recipient HLA could be determined from urine samples of kidney transplant recipients. Urinary DNA chimerism was found to be present following kidney transplantation [5]. A previous study by Srikantha et al. has retrospectively tissue typed the recipient urine to determine mismatched donor HLA specificities; however, their results revealed that over 8% (3/34) urine samples failed to produce adequate DNA for HLA typing without further resolution [6]. In the current study, with previous experience in modified PCR profile for allograft tissue [3,4], we are able to enhance the detection rate of the mismatched HLA antigens from urine samples in low quantity of DNA extracts.

The HLA typing data from the urine samples were expected to demonstrate a combination of both recipient and donor HLA phenotypes. Mismatched donor HLA could thus be identified from the urine result and the recipient's HLA data. We reason that the HLA typing obtained from urine can avoid the invasive procedure of allograft kidney biopsy in obtaining the same results. This methodology is thus a useful tool with the prospective application in the maintenance of an accurate patient sensitization history especially when transplant performed elsewhere or tissue typing were incomplete or less precise and may also abet post-transplant immunological monitoring [7,8].

## Materials and Methods

### Patients

Between May and December in 2012, 155 fresh early morning urine samples were collected from kidney transplant recipients with known donor-recipient HLA phenotypes from three renal transplant centers in Hong Kong. The study was approved by the Institutional Review Board of the University of Hong Kong/Hospital Authority Hong Kong West Cluster (HKU/HA HKW IRB) in accordance with the Declaration of Helsinki (Reference No. UW11-394). Samples were collected after written informed consent was granted. If the participants are incompetent in giving consent, legal guardians or legally authorized representatives were consented on the behalf of the participants. In order to minimize cellular degradation after collection, the urine samples were delivered to the HLA laboratory for DNA extraction within 4 hours. The extracted DNA from urine samples were kept at -30°C for storage before further testing.

### DNA extraction

Fifty milliliter of fresh early morning urine was collected and centrifuged at 3,600 rpm for 30 minutes. The supernatant was discarded and the sediment was collected and washed with 1X phosphate buffered saline and followed by centrifugation at full speed for 10 minutes. These steps were repeated twice. DNA extraction was performed by automatic EZmag Genomic DNA Whole Blood Kit (Texas BioGene Inc., Taiwan). The DNA extracts were kept at -30°C before use. Among the 155 DNA extracts, 35 representative samples covering all possible ranges of DNA yields were further validated by polymerase chain reaction-specific sequence primers (PCR-SSP) in which the composition of reaction mixture as well as the PCR profile for each cycle were utilized as described in the manufacturer’s manual (Collaborative Transplant Study, CTS, Department of Transplantation Immunology, University Clinic Heidelberg, Germany) 4[4] but the number of PCR cycles selection against loci was tailor-made according to the concentration of the DNA extracts as shown in Table 1. In brief, 25 μL and 50 μL DNA samples were added to 69 μL and 138 μL 7.5% CTS PCR buffer for HLA-A and B loci respectively, whereas 25 μL DNA was added to 69 μL 5.0% CTS master mix for HLA-DRB1 loci. In addition, hot start JumpStart *Taq* DNA polymerase (Sigma, USA), 0.4 units/well was used to enhance the sensitivity and specificity of the reaction. Ten microliters reaction mixtures were dispensed into each tray well containing specific lyophilized primer mixes and then incubated at 94°C for 2 min to activate the JumpStart *Taq* DNA polymerase, followed by two subsequent temperature cycling depend on various DNA yields at first cycle at 94°C for 15 sec and 65°C for 1 min; and then second cycle at 94°C for 15 sec, 61°C for 50 sec and 72°C for 30 sec. PCR product is kept at 4°C and were examined under gel electrophoresis through 2% agarose gel in 1X TAE.

**Table 1:** Thermal cycling profile selection for samples with different DNA amount.

HLA loci	Amount of DNA from urine samples (ng)			
	250–1,000	1,000–2,000	2,000–5,000	>5,000
	Number of cycle in two-step PCR reaction^a^			
	(annealing temperature: 65°C, 61°C)			
A	50, 50	20, 50	20, 40	
B		30, 50		20, 40
DRB1				

^a^Adoption of two step PCR reaction in order to enhance template quantity and end product amplification.

## Results

Among the 155 urine samples, 34.8% (54/155), 25.8% (40/155), 18.1% (28/155) and 21.3% (33/155) yielded DNA amount >5,000 ng, 2,000–5,000 ng, 1,000–2,000 ng and 250–1,000 ng respectively with purity (A_260_/A_280_) of 1.7–1.9. The time interval of specimen collection after transplantation ranged from 349–7,125 days, 110–6,451 days, 14–3,850 days and 397–3,520 days respectively (Table 2). The data showed no correlation between DNA quantity in urine samples and the time elapsed since transplantation.

**Table 2:** Amount of DNA obtained from urine samples and the time interval of specimen collection after transplantation.

Amount of DNA from urine samples (ng)	250–1,000	1,000–2,000	2,000–5,000	>5,000
Percentage of samples	21.3%	18.1%	25.8%	34.8%
	(33/155)	(28/155)	(40/155)	(54/155)
Time after transplantation (days)	397–3,520	14–3,850	110–6,451	349–7,125

Trials have been taken to adjust the number of cycles of PCR to obtain the optimal amplification results in particular for those low DNA concentrations (data not shown). In the trials, serial dilution of DNA from 1,000 to 125 ng was examined by employing the 100-cycle PCR profile (i.e. by employing two subsequent 50-cycle PCR at annealing temperature 65°C and 61°C in order to enhance template quantity and end product amplification). The experiment results revealed that the end-point sensitivity of the protocol could be up to 250 ng for HLA-A, HLA-B and HLA-DRB1 loci. All urine samples can yield adequate DNA quantity and quality required for HLA typing.

The impact of *Escherichia coli* colonization in the urine samples on this protocol was also investigated; it was proved that the microbial DNA of *E*. *coli* caused no interference on the accuracy of HLA typing of the patients. On the other hand, the microbial protein contaminant could be minimized by repeat washing and centrifuging the urine sediment with phosphate buffer as well as buffer washing during DNA extraction. The low protein contamination was indicated by the purity range in A_260_/A_280_ of 1.7–1.9 in all DNA extracts.

**Fig 1:**
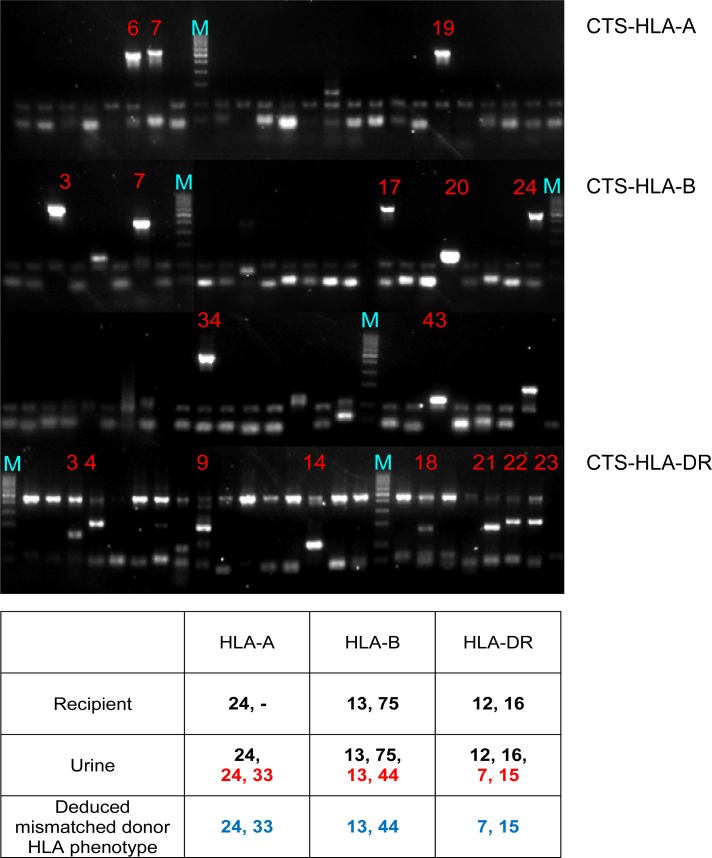
A representative PCR-SSP gel image of HLA-A, -B, and -DR loci of a urine sample from a kidney transplant patient. Lanes 6, 7 and 19 of HLA-A loci showed positive amplifications of A24 and A33 antigens. Lanes 3, 7, 17, 20, 24, 34 and 43 of HLA-B loci showed positive amplifications of B13, B44 and B75. Lanes 3, 4, 9, 14, 18, 21, 22 and 23 of HLA-DR loci showed positive amplifications of DR7, DR12, DR15 and DR16. M: Bio-ladder 50–1,000 bp (Biosynthesis, Texas, United States).

To study the practicability of this protocol for deducing donor HLA typing from a wide range of DNA level extracted from urine samples, 35 representative DNA samples that comprised 10, 10, 10 and 5 samples with total DNA yield in the ranges of 5,500–24,000 ng, 2,150–4,900 ng, 1,200–1,900 ng and 620–950 ng respectively; were further tested by employing the molecular protocol. The HLA phenotypes of the donor-recipient pairs were compared (Tables 3, 4, 5 and 6). All the HLA data from the samples successfully represented a composite of both patient and donor HLA phenotypes (35/35). With no drop out, 31, 48 and 51 mismatched donor HLA phenotypes bolded in Table 3 to Table 6 were detected at HLA-A, -B and -DRB1 loci, respectively. The PCR-SSP gel image of a representative sample was shown in Fig 1. Antigen assignments were based on the reaction patterns provided by the manufacturer.

**Table 3:** HLA typing results of the five recipient-donor pairs and recipient urine samples with DNA yield 250 ng– 1,000 ng.

	HLA											
	A				B				DR			
Patient 1 (Whole blood)	2	/	/	33	/	46	/	58	/	9	13	/
Donor (Whole blood)	/	**11**	**26**	/	**13**	/	**48**	/	**8**	/	/	**15**
Patient urine	2	**11**	**26**	33	**13**	46	**48**	58	**8**	9	13	**15**
Mismatches deduced	/	**11**	**26**	/	**13**	/	**48**	/	**8**	/	/	**15**
Patient 2 (Whole blood)	2	30	/	/	13	46	/	/	7	/	15	/
Donor (Whole blood)	2	/	/	/	/	46	**75**	/	/	**9**	15	/
Patient urine	2	30	/	/	13	46	**75**	/	7	**9**	15	/
Mismatches deduced	/	/	/	/	/	/	**75**	/	/	**9**	/	/
Patient 3 (Whole blood)	24	/	/	/	35	51	/	/	4	/	11	/
Donor (Whole blood)	/	**33**	/	/	35	/	**61**	/	/	**8**	/	**14**
Patient urine	24	**33**	/	/	35	51	**61**	/	4	**8**	11	**14**
Mismatches deduced	/	**33**	/	/	/	/	**61**	/	/	**8**	/	**14**
Patient 4 (Whole blood)	2	/	33	/	17	46	/	/	/	/	14	17
Donor (Whole blood)	/	**26**	33	/	17	/	**60**	/	**3**	**12**	/	/
Patient urine	2	**26**	33	/	17	46	**60**	/	**3**	**12**	14	17
Mismatches deduced	/	**26**	/	/	/	/	**60**	/	**3**	**12**	/	/
Patient 5 (Whole blood)	2	11	/	/	/	46	48	/	/	9	15	/
Donor (Whole blood)	/	11	**30**	/	**13**	46	/	/	**7**	9	/	/
Patient urine	2	11	**30**	/	**13**	46	48	/	**7**	9	15	/
Mismatches deduced	/	/	**30**	/	**13**	/	/	/	**7**	/	/	/

Mismatched donor HLA phenotypes are shown in **bold**.

**Table 4:** HLA typing results of the ten recipient-donor pairs and recipient urine samples with DNA yield between 1,000 ng and 2,000 ng.

	HLA											
	A				B				DR			
Patient 6 (Whole blood)	2	11	/	/	/	51	/	/	9	15	/	/
Donor (Whole blood)	/	/	**24**	/	**13**	51	/	/	9	15	/	/
Patient urine	2	11	**24**	/	**13**	51	/	/	9	15	/	/
Mismatches deduced	/	/	**24**	/	**13**	/	/	/	/	/	/	/
Patient 7 (Whole blood)	11	/	/	/	/	75	/	/	/	12	14	/
Donor (Whole blood)	/	**24**	**26**	/	**46**	75	/	/	**9**	12	/	/
Patient urine	11	**24**	**26**	/	**46**	75	/	/	**9**	12	14	/
Mismatches deduced	/	**24**	**26**	/	**46**	/	/	/	**9**	/	/	/
Patient 8 (Whole blood)	2	/	33	/	/	40	/	/	/	9	/	/
Donor (Whole blood)	2	**24**	/	/	**22**	/	**46**	/	**4**	/	**14**	/
Patient urine	2	**24**	33	/	**22**	40	**46**	/	**4**	9	**14**	/
Mismatches deduced	/	**24**	/	/	**22**	/	**46**	/	**4**	/	**14**	/
Patient 9 (Whole blood)	2	11	/	/	5	/	/	40	/	4	9	/
Donor (Whole blood)	/	11	**33**	/	/	**17**	**35**	/	**3**	/	/	**12**
Patient urine	2	11	**33**	/	5	**17**	**35**	40	**3**	4	9	**12**
Mismatches deduced	/	/	**33**	/	/	**17**	**35**	/	**3**	/	/	**12**
Patient 10 (Whole blood)	1	11	/	/	37	/	/	75	/	10	/	15
Donor (Whole blood)	/	/	**24**	**29**	/	**39**	**60**	60	**4**	/	**13**	/
Patient urine	1	11	**24**	**29**	37	**39**	**60**	75	**4**	10	**13**	15
Mismatches deduced	/	/	**24**	**29**	/	**39**	**60**	/	**4**	/	**13**	/
Patient 11 (Whole blood)	11	/	/	/	/	60	75	/	9	/	12	/
Donor (Whole blood)	11	**29**	/	/	**7**	60	/	/	9	**10**	/	/
Patient urine	11	**29**	/	/	**7**	60	75	/	9	**10**	12	/
Mismatches deduced	/	**29**	/	/	**7**	/	/	/	/	**10**	/	/
Patient 12 (Whole blood)	2	11	/	/	/	58	62	/	/	11	/	17
Donor (Whole blood)	/	11	**33**	/	**13**	58	/	/	**9**	/	**14**	/
Patient urine	2	11	**33**	/	**13**	58	62	/	**9**	11	**14**	17
Mismatches deduced	/	/	**33**	/	**13**	/	/	/	**9**	/	**14**	/
Patient 13 (Whole blood)	2	/	26	/	/	/	60	/	4	8	/	/
Donor (Whole blood)	2	**24**	/	/	**39**	**46**	/	/	4	/	**14**	/
Patient urine	2	**24**	26	/	**39**	**46**	/	/	4	8	**14**	/
Mismatches deduced	/	**24**	/	/	**39**	**46**	/	/	/	/	**14**	/
Patient 14 (Whole blood)	/	24	29	/	7	/	54	/	10	/	15	/
Donor (Whole blood)	**11**	/	/	**33**	/	**46**	/	/	/	**12**	15	/
Patient urine	**11**	24	29	**33**	7	**46**	54	/	10	**12**	15	/
Mismatches deduced	**11**	/	/	**33**	/	**46**	/	/	**12**	/	/
Patient 15 (Whole blood)	2	11	/	/	46	/	62	/	4	12	/	/
Donor (Whole blood)	2	11	/	/	/	**54**	/	**75**	4	/	**15**	/
Patient urine	2	11	/	/	/	**54**	/	**75**	/	/	**15**	/
Mismatches deduced	/	/	/	/	/	**54**	/	**75**	/	/	**15**	/

Mismatched donor HLA phenotypes are shown in **bold**.

**Table 5:** HLA typing results of the ten recipient-donor pairs and recipient urine samples with DNA yield between 2,000 ng and 5,000 ng.

	HLA											
	A				B				DR			
Patient 16 (Whole blood)	11	24	/	/	13	35	/	/	/	/	14	15
Donor (Whole blood)	11	/	/	/	/	/	**60**	**75**	**4**	**9**	/	/
Patient urine	11	24	/	/	13	35	**60**	**75**	**4**	**9**	14	15
Mismatches deduced	/	/	/	/	/	/	**60**	**75**	**4**	**9**	/	/
Patient 17 (Whole blood)	/	29	33	/	7	58	/	/	4	/	15	/
Donor (Whole blood)	**11**	/	33	/	/	58	/	/	/	**13**	/	**17**
Patient urine	**11**	29	33	/	/	/	/	/	4	**13**	15	**17**
Mismatches deduced	**11**	/	/	/	/	/	/	/	/	**13**	/	**17**
Patient 18 (Whole blood)	11	/	/	/	/	55	65	/	11	/	15	/
Donor (Whole blood)	11	/	/	/	**46**	/	/	**75**	/	**12**	15	/
Patient urine	11	/	/	/	**46**	55	65	**75**	11	**12**	15	/
Mismatches deduced	/	/	/	/	**46**	/	/	**75**	/	**12**	/	/
Patient 19 (Whole blood)	11	/	33	/	/	51	58	/	9	/	13	/
Donor (Whole blood)	11	**24**	/	/	**48**	51	/	/	9	**12**	/	/
Patient urine	11	**24**	33	/	**48**	51	58	/	9	**12**	13	/
Mismatches deduced	/	**24**	/	/	**48**	/	/	/	/	**12**	/	/
Patient 20 (Whole blood)	/	24	33	/	54	58	/	/	/	/	9	/
Donor (Whole blood)	**2**	/	/	/	/	/	**60**	**71**	**4**	**8**	/	/
Patient urine	**2**	24	33	/	54	58	**60**	**71**	**4**	**8**	9	/
Mismatches deduced	**2**	/	/	/	/	/	**60**	**71**	**4**	**8**	/	/
Patient 21 (Whole blood)	2	/	/	26	8	/	38	/	4	/	/	17
Donor (Whole blood)	/	**11**	**24**	/	/	**13**	/	**58**	/	**14**	**15**	/
Patient urine	2	**11**	**24**	26	8	**13**	38	**58**	4	**14**	**15**	17
Mismatches deduced	/	**11**	**24**	/	/	**13**	/	**58**	/	**14**	**15**	/
Patient 22 (Whole blood)	/	11	/	/	/	/	/	75	/	12	/	/
Donor (Whole blood)	**2**	/	**33**	/	/	**48**	**58**	/	**11**	/	**17**	/
Patient urine	**2**	11	**33**	/	/	**48**	**58**	75	**11**	12	**17**	/
Mismatches deduced	**2**	/	**33**	/	/	**48**	**58**	/	**11**	/	**17**	/
Patient 23 (Whole blood)	2	/	24	/	/	13	/	61	8	/	12	/
Donor (Whole blood)	2	**11**	/	/	/	13	**46**	/	/	**11**	/	**15**
Patient urine	2	**11**	24	/	/	13	**46**	61	8	**11**	12	**15**
Mismatches deduced	/	**11**	/	/	/	/	**46**	/	/	**11**	/	**15**
Patient 24 (Whole blood)	24	/	/	/	/	13	/	75	/	12	/	16
Donor (Whole blood)	24	**33**	/	/	/	13	**44**	/	**7**	/	**15**	/
Patient urine	/	**33**	/	/	/	13	**44**	/	**7**	12	**15**	16
Mismatches deduced	/	**33**	/	/	/	/	**44**	/	**7**	/	**15**	/
Patient 25 (Whole blood)	2	11	/	/	13	46	/	/	/	15	17	/
Donor (Whole blood)	2	11	/	/	/	/	**54**	**75**	**4**	15	/	/
Patient urine	2	11	/	/	13	46	**54**	**75**	**4**	15	17	/
Mismatches deduced	/	/	/	/	/	/	**54**	**75**	**4**	/	/	/

Mismatched donor HLA phenotypes are shown in **bold**.

**Table 6:** HLA typing results of the ten recipient-donor pairs and recipient urine samples with DNA yield > 5,000 ng.

	HLA											
	A				B				DR			
Patient 26 (Whole blood)	24	33	/	/	13	44	/	/	/	/	13	15
Donor (Whole blood)	24	/	/	/	13	/	**54**	/	**9**	**12**	/	/
Patient urine	24	33	/	/	13	44	**54**	/	**9**	**12**	13	15
Mismatches deduced	/	/	/	/	/	/	**54**	/	**9**	**12**	/	/
Patient 27 (Whole blood)	2	11	/	/	13	56	/	/	/	15	/	/
Donor (Whole blood)	/	11	**24**	/	13	/	**58**	/	**14**	/	**17**	/
Patient urine	2	11	**24**	/	13	56	**58**	/	**14**	15	**17**	/
Mismatches deduced	/	/	**24**	/	/	/	**58**	/	**14**	/	**17**	/
Patient 28 (Whole blood)	11	/	30	/	13	46	/	/	4	7	/	/
Donor (Whole blood)	11	**24**	/	/	/	46	**62**	/	4	/	**11**	/
Patient urine	11	**24**	30	/	13	46	**62**	/	4	7	**11**	/
Mismatches deduced	/	**24**	/	/	/	/	**62**	/	/	/	**11**	/
Patient 29 (Whole blood)	2	11	/	/	/	60	/	/	4	/	15	/
Donor (Whole blood)	2	11	/	/	**51**	/	**75**	/	4	**9**	/	/
Patient urine	2	11	/	/	**51**	60	**75**	/	4	**9**	15	/
Mismatches deduced	/	/	/	/	**51**	/	**75**	/	/	**9**	/	/
Patient 30 (Whole blood)	11	/	/	/	27	54	/	/	4	9	/	/
Donor (Whole blood)	11	**31**	/	/	/	/	**55**	**60**	4	/	**14**	/
Patient urine	11	**31**	/	/	27	54	**55**	**60**	4	9	**14**	/
Mismatches deduced	/	**31**	/	/	/	/	**55**	**60**	/	/	**14**	/
Patient 31 (Whole blood)	/	24	/	/	/	56	60	/	9	/	13	/
Donor (Whole blood)	**11**	/	/	/	**27**	/	60	/	9	**11**	/	/
Patient urine	**11**	24	/	/	**27**	56	60	/	9	**11**	13	/
Mismatches deduced	**11**	/	/	/	**27**	/	/	/	/	**11**	/	/
Patient 32 (Whole blood)	11	24	/	/	/	58	60	/	4	/	13	/
Donor (Whole blood)	11	/	/	/	**46**	/	/	**62**	4	**9**	/	/
Patient urine	11	24	/	/	**46**	58	60	**62**	4	**9**	13	/
Mismatches deduced	/	/	/	/	**46**	/	/	**62**	/	**9**	/	/
Patient 33 (Whole blood)	11	33	/	/	55	58	/	/	/	12	/	17
Donor (Whole blood)	11	33	/	/	/	58	**75**	/	**9**	/	**13**	/
Patient urine	11	33	/	/	55	58	**75**	/	**9**	12	**13**	17
Mismatches deduced	/	/	/	/	/	/	**75**	/	**9**	/	**13**	/
Patient 34 (Whole blood)	2	31	/	/	44	51	/	/	7	/	17	/
Donor (Whole blood)	2	/	**33**	/	44	/	**62**	/	7	**11**	/	/
Patient urine	2	31	**33**	/	44	51	**62**	/	7	**11**	17	/
Mismatches deduced	/	/	**33**	/	/	/	**62**	/	/	**11**	/	/
Patient 35 (Whole blood)	2	11	/	/	46	75	/	/	9	11	/	/
Donor (Whole blood)	2	/	/	/	46	/	/	/	9	/	**12**	/
Patient urine	2	11	/	/	46	75	/	/	9	11	**12**	/
Mismatches deduced	/	/	/	/	/	/	/	/	/	/	**12**	/

Mismatched donor HLA phenotypes are shown in **bold**.

## Discussion

The application of DNA extraction in urine sample and amplification by PCR-SSP in the diagnosis of acute rejection was reported by Zhang et al. [9,10]. In the current study, we used a hot-start DNA polymerase to enhance the sensitivity and specificity of PCR and the amplification profile was optimized and tailor-made according to different yield of DNA extracts as well as HLA loci (Table 1). The correct allele-specific fragments could be distinguished from non-specific PCR products by different band size as described in the manufacturer’s manual. Weak false positive reactions due to addition amplification cycles were very rarely encountered, however, they could be interpreted as very rare HLA alleles with no impact on the final HLA phenotype interpretation if occur. In this study, 130 mismatched donor HLA antigens could be deduced in PCR-SSP tested in 35 urine samples containing a wide range of DNA yield and time interval after transplantation. The yield of DNA did not correlate with the time interval after transplantation.

Although our results demonstrated correct mismatched antigens assignments of both patient and donor HLA of 35 urine samples in our present cohort, the impact of preferential PCR amplification of one allele over another in a heterozygous sample should be neglected. For PCR-based HLA typing in which amplicons differ in size for different alleles, preferential amplification of the shorter allele product may occur [11]. As the tested urine samples contained both recipient and donor DNAs, the occurrence of preferential amplification may hamper the accurate HLA assignments. The donor-derived DNA may exist in minor proportion of the urine DNA extract, thus preferential amplification of the major portion of the DNA allele may occur. In such case, amplification of one or more alleles may fail leading to allele dropout. To assess the performance of the modified protocol in order to rule out the occurrence of such scenario, testing serial dilution of mixed DNAs from the donor and recipients would be desirable. However, due to the unavailability of donor DNA and limited DNA extract from urine of each mismatched HLA typing pairs, such validation was not feasible in our current experimental setting. Further experiments will be conducted by artificially mixing two individual urine DNA extracts.

The presence of DSA is important in diagnosing antibody-mediated rejection, predicting graft survival and assessing response to rejection treatment. Serial monitoring of DSA allows early intervention which may have significant impact on long-term allograft survival [7]. This application is of particular concern in our local clinical setting as more than two-third of local kidney transplant recipients received their grafts outside Hong Kong [12,13]. For this group of patients the donor HLA phenotype is often not known, hence it was not possible to detect the emergence of DSA after transplantation.

This study has demonstrated that this non-invasive assay has conceivable application for the detection of mismatched donor HLA in kidney transplant recipients with unknown or incomplete information of donor HLA phenotype. In addition, it can also utilize urine samples of transplant recipients to generate their respective historical HLA typing information of the donor and thereby facilitating post-transplant immunological monitoring [8]. It is also useful when the transplant was performed long ago when tissue typing was less precise or those patients have failed graft function and required re-transplant. Without the precise donor’s HLA typing, it is not feasible to identify DSA and assigning unacceptable mismatched antigens for patients who require re-transplant, which may affect the survival of the new allograft. The application of DNA extraction in urine samples demonstrated in our current protocol offers a non-invasive alternative with superior sensitivity and circumvents the necessity of an invasive allograft biopsy procedure. This attractive diagnostic method would be welcomed by renal physician for management of post-transplant renal patients with insufficient donor information who underwent transplantation overseas.
